# Bioecological profiles of preschool children’s individual, familial, and educational characteristics, and their relations with school adjustment, academic performance, and executive function in first grade

**DOI:** 10.3389/fpsyg.2023.1185098

**Published:** 2023-07-05

**Authors:** Young-Eun Lee

**Affiliations:** Department of Early Childhood Education, Gachon University, Seongnam, Republic of Korea

**Keywords:** academic performance, bioecological profiles, educational environment, executive function, familial environment, individual characteristics, school adjustment

## Abstract

This study investigates the relationships between distinct bioecological profiles of individual, familial, and educational characteristics of preschool children and their school adjustment, academic performance, and executive function in first grade. Data on 11 indicators of personal and environmental characteristics were collected from 1,016 five-year-old Korean preschoolers using a national-level open dataset. Latent profile analysis identified five profiles that were associated with different levels of school adjustment, academic performance, and executive function one year later when the preschoolers became first graders. The “Good Social Competence by Good Familial Environment” profile was the most associated with levels of school adjustment, academic performance, and executive function. The “Good Social Competence by Good Educational Environment” profile was more associated with levels of school adjustment and executive function than the “Moderate” profile but less associated with these levels than the “Good Social Competence by Good Familial Environment” profile. Findings indicate that the environment, rather than individual characteristics of preschoolers, plays a more significant role in their elementary school adjustment, academic performance, and executive function, and that their familial environment plays a more significant role than their educational environment. The study highlights the importance of creating supportive familial environments for preschool children to improve their school adjustment, academic performance, and executive function in elementary school, and provides a useful guide for practitioners and policymakers seeking to improve children’s academic and socioemotional outcomes.

## 1. Introduction

The individual and the environment influence children’s school adjustment and academic performance. According to the bioecological model, individual developmental characteristics and various social contexts influence children’s adjustment and development (Bronfenbrenner, 1976). The interactional relational model also emphasizes that children’s school adjustment and academic performance are impacted by the interaction between the familial and educational environment, as well as their individual gender and developmental status (Williams et al., 2019). These perspectives underscore the importance of examining how children’s school adjustment and academic performance are affected differently through the context of ongoing interactions with individual characteristics and environments. Thus, the current study constructed a latent profile group based on 11 indicators of individual, family, and education environments of Korean preschoolers, and examined how the profiles were associated with school adjustment, academic performance, and executive function in the first grade of elementary school.

### 1.1. School adjustment, academic performance, and executive function in the first year of elementary school

One of the major environmental changes that occur in life is children’s entrance to elementary school. This opens up a wider world of meeting new people and engaging in new activities (Bierman et al., 2008; Pears et al., 2015; Yelverton and Mashburn, 2018). In this period, in particular, Korean children experience some difficulties in adjusting to the educational environment, as it emphasizes group harmony and discipline, leading to stress from interpersonal relationships and adherence to rules (Kim and Park, 2005; Park, 2010). There is ample worldwide evidence that adjustment and academic performance in elementary school affect children’s current and future social–emotional skills and academic achievement (Heckman, 2008; Forget-Dubois et al., 2009; Brydges et al., 2012). Specifically, school adjustment is related to children’s prosocial behavior, emotional and behavioral regulation, literacy, and math skills (Heckman, 2008; Forget-Dubois et al., 2009). In addition, interindividual differences in elementary school adjustment can continue throughout middle and late childhood (Forget-Dubois et al., 2009; Kingdon et al., 2017; Lee et al., 2020; Sung, 2021). It has been reported, for example, that poor school adjustment and academic performance in elementary school can result in negative consequences in late childhood and adulthood, such as low high school graduation rates, low college enrollment rates, limited career choices, and mental/physical health problems in adulthood (Heckman, 2008; Murray and Farrington, 2010). Executive function refers to mental processes that help monitor and control thoughts and behaviors such as inhibitory control, working memory, planning, and cognitive flexibility (Diamond, 2013). These begin to appear from the first year of life, and children’s interindividual differences become increasingly evident at school age (Huizinga et al., 2006; Wu et al., 2011). The level of executive function in elementary school has been found to be associated with later adaptive behavior, social cognitive understanding, and moral ability, as well as academic achievement (Kochanska et al., 1996; Carlson and Moses, 2001; Bull et al., 2011; Brydges et al., 2012). Therefore, considering the evident longitudinal influence of school adjustment, academic performance, and executive function in the first grade of elementary school, it is necessary to specifically investigate the factors that affect them.

### 1.2. Individual characteristics affecting school adjustment, academic performance, and executive function in the first year of elementary school

Previous studies have reported that socio-emotional individual characteristics, such as peer play interactions and self-esteem, are important factors affecting the school adjustment and academic performance of elementary school students (Sturge-Apple et al., 2010; Williams et al., 2019). Specifically, children who were more receptive to others’ emotional perspectives and identified situations that could trigger different emotional responses were more comfortable adjusting to school (O'Connor et al., 2014). Those who played cooperatively with their peers had higher quality relationships with their teachers after one year, and their better social skills to listen in class positively affected school adjustment and academic performance (Legkauskas and Magelinskaite-Legkauskiene, 2019). Self-esteem is also an important predictor of peer relationships, and low self-esteem led to school adjustment problems (Wang et al., 2016; In-Albon et al., 2017; Xin et al., 2019). A large body of research has investigated the associations between self-esteem and school adjustment for adolescents. However, the current study examines preschoolers’ self-esteem as an emotional index of individual characteristics and its effect on first-grade school adjustment.

### 1.3. Familial characteristics affecting school adjustment, academic performance, and executive function in the first year of elementary school

Previous studies have established that family environment, such as parenting behavior, parent–child interaction, and family interaction, are important predictors of school adjustment and academic performance (Sturge-Apple et al., 2010; Pears et al., 2015). Specifically, positive parenting behavior is a key element of school readiness and later academic outcomes, as well as a significant component of school-readiness programs such as Head Start (Kiernan and Mensah, 2011; Pears et al., 2015). However, negative parenting, such as lack of parental involvement or parental insensitivity, compromises children’s school-readiness skills (Kim et al., 2022; Oh et al., 2022). Children who also displayed demoralization, inconsistency, less responsiveness, provocativeness, and harsh behaviors were less able to adapt to school (Chazan-Cohen et al., 2009; Okado et al., 2014). Evans et al. (2013) determined that cumulative family risk, rather than any single family risk, had a more detrimental effect on children’s academic performance. According to Iruka et al. (2012), the success or failure of Asian children’s school adjustment may be influenced by family characteristics (e.g., focus on family duties) rather than parenting. As a country with a collectivistic and relationship-centered culture that emphasizes “connection with family,” South Korea has high expectations for children’s school adjustment, especially in terms of academic achievement (Tamis-LeMonda et al., 2008, p. 187; Karam et al., 2013; Woo and Hodges, 2015; Kim and Chung, 2017). Therefore, it is necessary to examine how family characteristics such as cohesion and flexibility affect school adjustment and academic performance, along with parenting behavior and parent–child interaction.

### 1.4. Educational characteristics affecting school adjustment, academic performance, and executive function in the first year of elementary school

Preschool environments are significant predictors of school adjustment and academic performance (Raver et al., 2011; Yoshikawa et al., 2013). Empirical studies have indicated that higher levels of teacher interaction with preschoolers are associated with their competencies in mathematics and literacy, such as receptive and expressive vocabulary, and reading and writing skills (Keys et al., 2013; Hatfield et al., 2016). Lower levels of such interaction are associated with less empathy, inhibitory control, problem-solving, and more disruptive behavior (Siekkinen et al., 2013; Hatfield et al., 2016). When teachers ask open-ended questions through high-quality interactions and promote learning through problem-solving, practical application, and language modeling, they improve preschoolers’ memory and attention, and consequently enhance their overall executive functioning (Diamond et al., 2007). In addition, teachers who believe that they can make a difference in children’s achievement exhibit more support and provide a more positive classroom environment, thereby resulting in strong literacy skills in children (Guo et al., 2012). A meta-study also found that, despite a small effect size, teachers’ self-efficacy was related to children’s school adjustment and achievement (Zee and Koomen, 2016). The well-defined setting of the classroom environment also contributes to children’s interactive behavior during play, school adjustment, and academic performance (Cunningham, 2010; Abbas et al., 2012; Vitiello et al., 2012).

### 1.5. Present study

This study applied a person-centered approach to examine the various compositions of the bioecological environment of preschoolers. Three different dimensions (individual characteristics, family environment, and educational environment) were used to determine whether profiles could be identified. A total of 11 indicators across the three dimensions include individual characteristics (play interaction, play disruption, play disconnection, and self-esteem), family environment (warm parenting, parent–child interaction, balanced family cohesion, and balanced family flexibility), and educational environment (classroom environment, teacher-child interaction, and teacher self-efficacy). Although this was an exploratory study, eight distinct profiles of preschoolers were expected, representing cases where the level of the bioecological environment varied by domain (e.g., preschoolers with high social competence in poor family environments and good educational environments). This study also investigated whether the distinct profiles of the bioecological environment were associated with school adjustment, academic achievement, and executive function in the first grade. After controlling for children’s demographic risk factors (e.g., child gender, maternal education level, family income), profiles with greater bioecological support were expected to show higher levels of school adjustment, academic achievement, and executive function in the first grade.

## 2. Methods

### 2.1. Participants

Data were collected from the Panel Study on Korean Children (PSKC) conducted by the Korean Institute of Child Care and Education (KICCE). As a nationally representative longitudinal study, the PSKC has annually collected comprehensive information on children’s ecological environments, including characteristics of children, parents, families, schools, and local communities, since 2008. Using a stratified multi-stage sampling method, the PSKC recruited a total of 2,150 children born between April and July 2008. The present study used the 7th and 8th waves of data obtained in 2014, one year before children entered school (Time1), and 2015, when they entered elementary school (Time2). The sampling attrition rate of the PSKC was 24.7% by 2014. Among the maintained sample of 1,620 children, cases that required special education and did not have nine or more indicators for latent profile analysis (LPA) were excluded. Hence, the final analytic sample consisted of 1,016 children. This study was approved by the Gachon University Institutional Review Board (#1044396-202304-HR-048-01). Written informed consent was obtained from each participant at the time of recruitment by the Korea Institute of Child Care and Education. In 2014, the average age of the children (51% boys) was 87.78 months (*SD* = 1.45). Most parents in this sample were in their 30s (fathers: M_age_ = 39.26, *SD* = 4.02 and mothers: M_age_ = 36.82, *SD* = 3.71). The average age of preschool teachers was 32.26 years (*SD* = 7.86). In terms of educational attainment, 498 (49%) fathers and 399 (39%) mothers held 4-year college degrees or higher, 216 (21%) fathers and 306 (30%) mothers held 2- or 3-year college degrees, while 293 (29%) fathers and 308 (30%) mothers had high school diplomas or lower educational degrees. A total of 52% of preschool teachers (*n* = 452) had 4-year college degrees or higher, 43% of teachers (*n* = 370) had 2- or 3-year college degrees, while 5% (*n* = 45) had high school diplomas or cyber university degrees. In terms of parental employment status, 909 (96%) fathers and 475 (47%) mothers were employed. The average family income per month was 4,410,000 Korean Won (approx. US $3,257) (*SD* = 195.48).

### 2.2. Measurements

#### 2.2.1. Measurements of child characteristics

This study adopted two measurements to assess four indicators of children’s socio-emotional characteristics. The Penn Interactive Peer Play Scale was used to examine the peer play interactions of preschool children (Fantuzzo et al., 1998). Play interaction (1st indicator) comprised nine items (*α* = 0.79) reflecting creative, cooperative, and helpful behaviors that facilitate successful peer play interactions (e.g., “child helps to resolve conflicts between friends”). Play disruption (2nd indicator) comprised 13 items (*α* = 0.86) describing children’s aggressive and antisocial play behaviors (e.g., “child does not accept what friends suggest about play”). Play disconnection (3rd indicator) comprised eight items (*α* = 0.89) capturing withdrawn and avoidant behaviors that impede active participation in play with peers (e.g., “child is rejected by another friend”). Preschool teachers responded on a 4-point Likert scale ranging from “1 = not at all true” to “4 = always true.” Mean scores were calculated, with higher scores indicating greater cooperative peer play interaction, disruption, and disconnection, respectively. The Pictorial Scale of Perceived Competence and Social Acceptance was used to measure preschool children’s self-esteem (Harter and Pike, 1984). Self-esteem (4th indicator) comprised 25 items (*α* = 0.63) reflecting their perceived physical and cognitive competence and peer and maternal acceptance. Preschool children were presented with a picture plate and asked to identify the child that they were most like. They responded on a 4-point scale ranging from “1 = a little bit like that child” to “4 = a lot like that child.” Mean scores were calculated, with higher scores indicating greater self-esteem.

#### 2.2.2. Measurements of familial environments

Three measurements were used to assess four indicators of familial environments. The Korean Parenting Style Scale was used to examine parents’ warm parenting behavior. Warm parenting (5th indicator) comprised six items (*α* = 0.87) reflecting warmth in parenting through love and respect toward their children, intimacy, and communication. Mothers responded on a 5-point Likert scale ranging from “1 = not at all” to “5 = a lot.” Mean scores were calculated, with higher scores indicating warm parenting behavior. A modified version of the Home Environment, Activities, and Cognitive Stimulation from the Early Childhood Longitudinal Study Kindergarten Cohort was used to assess parent–child interaction. Such interaction (6th indicator) comprised nine items (*α* = 0.84) such as “I read books to my child” and “I sing songs with my child.” Mothers responded on a 4-point Likert scale ranging from “1 = not at all” to “4 = every day.” Mean scores were calculated, with higher scores indicating a high frequency of parent–child interaction. The Family Adaptability and Cohesion Evaluation Scale was used to examine healthier familial environments characterized as having appropriate levels of closeness and flexibility in the family (Olson, 2011). Balanced family cohesion (7th indicator) comprised seven items (*α* = 0.87) describing emotional bonding among family members. Balanced family flexibility (8th indicator) also comprised seven items (*α* = 0.81) capturing the quality and expression of leadership and organization, role relationship, and relationship rules and negotiations. Mothers responded on a 5-point Likert scale ranging from “1 = strongly disagree” to “5 = strongly agree.” Mean scores were calculated, with higher scores indicating more balanced and functional family interaction.

#### 2.2.3. Measurements of preschool educational environments

Three measurements were used to assess three indicators of educational environments. Developed by Seo et al. (2009), the Scale for Evaluation and Accreditation of Preschool Facilities was used to examine the preschool classroom environment. The classroom environment (9th indicator) comprised four items (*α* = 0.90), including “The space in the classroom was arranged considering the age, interest, and developmental characteristics of preschool children” and “There is enough material in the classroom to be used by preschool children when they want.” Teachers responded on a 5-point Likert scale ranging from “1 = not at all” to “5 = a lot.” Mean scores were calculated, with higher scores indicating a developmentally appropriate classroom environment. The Early Childhood Observation Instrument (Holloway and Reichhart-Erickson, 1988) was used to assess teacher-child interaction. This interaction (10th indicator) comprised six items (*α* = 0.87), such as “I interact frequently with children, showing affection and support” and “I encourage independence in children as they are ready.” Teachers responded on a 5-point Likert scale ranging from “1 = strongly disagree” to “5 = strongly agree.” Mean scores were calculated, with higher scores indicating a high quality of teacher-child interaction. Instructional self-efficacy of teachers was measured using the Teacher Self-efficacy Scale (Kim and Kim, 2008). Teacher self-efficacy (11th indicator) comprised seven items (*α* = 0.86), reflecting a teacher’s belief that their behavior can induce learning even when children are difficult or unmotivated. Teachers responded on a 5-point Likert scale ranging from “1 = strongly disagree” to “5 = strongly agree.” Mean scores were calculated, with higher scores indicating greater self-efficacy in teachers.

#### 2.2.4. Measurements of school adjustment, academic performance, and executive function

School adjustment in the first grade was measured using the School Adjustment Inventory, developed by Chi and Jung (2006). It comprised 35 items (*α* = 0.97), such as “The student follows the teacher’s instructions” and “The student listens attentively in class.” Teachers responded on a 5-point Likert scale ranging from “1 = strongly disagree” to “5 = strongly agree.” Mean scores were calculated, with higher scores indicating appropriate adjustment in school. Academic performance in the first grade was measured using the scale developed by Rhee et al. (2010) for a longitudinal effect study of comprehensive childcare services at the Samsung Childcare Center. The academic performance scale comprised 10 items (*α* = 0.98), reflecting Korean language, mathematics, and overall competence in school performance. Teachers responded on a 5-point Likert scale ranging from “1 = not yet” to “5 = proficient.” Mean scores were calculated, with higher scores indicating high-quality academic performance. Children’s executive function was measured using the Executive Function Difficulty Screening Questionnaire (Song, 2014). It comprised 40 items (*α* = 0.97), reflecting children’s planning-organizing, behavior-emotional control, and attention-concentration difficulties. Teachers responded on a 5-point scale ranging from “1 = strongly disagree” to “5 = strongly agree.” The scores of all items were reversed, and mean scores were calculated. Higher scores indicate greater executive function.

#### 2.2.5. Covariate

Preschool children’s demographic information was used as statistical controls. These included the child’s gender (0 = girl, 1 = boy), maternal education level (ranging from 0 = uneducated to 7 = graduate school), and subjective family socioeconomic status (ranging from “1 = lowest” to “10 = highest”) (Son et al., 2013; Schmerse, 2020).

### 2.3. Statistical analysis

First, Mplus 8 was used to conduct Latent Profile Analysis (LPA) to identify profiles of preschool children based on their individual characteristics, familial environment, and educational environment (Muthen and Muthen, 2000). Full Information Maximum Likelihood estimates were employed to account for missing data in study variables. Out of the 11 indicators, missing data was found for 10 indicators, with the extent of missing data ranging from 2.85 to 14.67%. Theoretically, considering the case in which the level of the bioecological environment is different for each domain, eight profiles were expected. However, the following statistical analysis can determine the appropriate number of profiles. To determine the most appropriate number of profiles, model fit statistics were compared using information-based criteria, including the Akaike Information Criterion (AIC), the Bayesian Information Criterion (BIC), and the Sample-Size-Adjusted BIC (SABIC), as well as entropy values (Nylund et al., 2007). In general, models with lower AIC, BIC, and SABIC values are considered better solutions. Higher entropy values (ideally above 0.70) indicate more precise classification of individuals. Additionally, the Lo–Mendell–Rubin Likelihood Ratio Test (LMR-LRT) and the Vuong-Lo–Mendell–Rubin Likelihood Ratio Test (VLMR-LRT) were employed as fit indices. A significant value of *p* for the LMR-LRT and the VLMR-LRT (*p* < 0.05) suggests that a model with k profiles has a better fit than one with k - 1 profiles (Nylund et al., 2007). The BIC and VLMR-LRT are the most robust among model selection criteria and possess the strongest power to detect an accurate number of profiles with the given data (Tein et al., 2013). The final model is determined based on these indices, as well as theoretical background and conceptual meaning (Jung and Wickrama, 2008).

Second, a total of three multiple regression models were conducted to examine how preschool children’s membership in a particular profile was associated with their school adjustment, academic performance, and executive function in the first grade. Based on LPA results, after controlling for preschool children’s gender, maternal education level, and family income, profile membership was added to the regression models as dummy variables. A dummy coding system was employed to examine differences among the five profiles regarding children’s school adjustment, academic performance, and executive function. Each regression for the three outcomes was run twice, with “boy” serving as the reference group in the first equation, and the “Moderate” profile serving as the reference group in the second equation.

## 3. Results

### 3.1. Descriptive statistics

Means with standard deviations and bivariate correlations for all study variables are presented in Table 1. To investigate differences by gender, an independent t-test was conducted for all variables. Results indicated that girls reported higher cooperative play interaction in preschool (*t* = 3.67, *p* < 0.001), school adjustment (*t* = 8.58, *p* < 0.001), academic performance (*t* = 6.04, *p* < 0.001), and executive function in the first grade (*t* = 10.95, *p* < 0.001) than boys. Boys reported higher play disruption in preschool (*t* = −8.08, *p* < 0.001) than girls.

**Table 1:** Descriptive statistics and bivariate correlations between the study variables.

	1	2	3	4	5	6	7	8	9	10	11	12	13	14
1														
2	−0.151^***^													
3	−0.345^***^	0.539^***^												
4	0.056	−0.013	−0.071^*^											
5	0.031	0.011	−0.043	0.135^***^										
6	0.063	−0.020	−0.064	0.123^***^	0.445^***^									
7	0.045	−0.013	−0.033	0.085^**^	0.435^***^	0.265^***^								
8	0.058	−0.008	−0.034	0.130^***^	0.445^***^	0.269^***^	0.809^***^							
9	0.126^***^	−0.022	−0.095^**^	−0.095^**^	−0.056	−0.070^*^	−0.003	−0.007						
10	0.195^***^	−0.121^***^	−0.191^***^	−0.031	−0.037	−0.034	−0.017	−0.003	0.355^***^					
11	0.196^***^	−0.067^*^	−0.126^***^	−0.012	0.007	−0.017	−0.014	−0.025	0.342^***^	0.660^***^				
12	0.285^***^	−0.214^***^	−0.213^***^	0.078^*^	0.069^*^	0.062	0.079^*^	0.113^***^	0.008	0.011	0.045			
13	0.200^***^	−0.111^**^	−0.228^***^	0.135^***^	0.096^**^	0.067^*^	0.087^*^	0.094^**^	−0.006	−0.010	0.011	0.575^***^		
14	0.281^***^	−0.349^***^	−0.247^***^	0.079^*^	0.057	−0.004	0.078^*^	0.083^**^	0.002	0.047	0.032	0.723^***^	0.612^***^	
*M*	3.09	2.12	1.86	3.00	3.63	2.26	3.96	3.58	4.28	4.23	3.90	3.96	4.24	2.69
SD	0.44	0.44	0.44	0.27	0.55	0.51	0.54	0.56	0.67	0.53	0.49	0.71	0.90	0.40
Skewness	−0.62	0.70	1.07	−0.58	−0.40	0.64	−1.02	−0.68	−1.40	−0.32	0.19	−0.72	−1.28	−1.83
Kurtosis	1.10	0.43	1.24	0.45	1.10	0.91	3.22	1.19	3.88	−0.35	−0.02	0.21	1.25	2.97
*N*	867	867	867	976	987	987	987	987	867	867	867	1,016	1,016	1,016

1 = Play interaction, 2 = Play disruption, 3 = Play disconnection, 4 = Self-esteem, 5 = Warm parenting, 6 = Parent–child interaction, 7 = Balanced family cohesion, 8 = Balanced family flexibility, 9 = Classroom environment, 10 = Teacher–child interaction, 11 = Teacher self-efficacy, 12 = School adjustment, 13 = Academic performance, 14 = Executive function. ^*^p < 0.05, ^**^p < 0.01, and ^***^p < 0.001.

### 3.2. Profile selection of the latent profile analyses

To identify the most appropriate number of profiles, five models with two to six profiles were estimated. The 5-profile model was found to be the optimal choice. As presented in Table 2, AIC, BIC, and sample-size adjusted-BIC (SABIC) continued to decrease with the addition of profiles. Entropy values fluctuated slightly with an increasing number of profiles. The 2- and 5-profile models exhibited higher classification accuracy than the other models. The *value of p* for the LMR-LRT remained significant for all solutions except the 6-profile model. Compared to the 2-profile model, the 5-profile model contained significantly different profiles from the previous profile, although the minimum proportion was 4%, which is less than 5%. In terms of fit statistics and the substantive interpretability of profiles, the 5-profile model was the most appropriate. Thus, this was selected as the final model.

**Table 2:** Fit indices for latent profile solutions.

Number of profiles	LL	Entropy	AIC	BIC	SABIC	LMR-LRT (*p* value)	VLMR-LRT (*p* value)	Class composition (% based on estimated model)
2 Profiles	−6,627.840	0.849	13,323.679	13,491.083	13,383.096	752.889 (*p* = 0.000)	761.951 (*p* = 0.000)	84%; 16%
3 Profiles	−6,388.042	0.700	12,868.085	13,094.572	12,948.472	473.891 (*p* = 0.009)	479.595 (*p* = 0.009)	47%; 37%; 16%
4 Profiles	−6,210.021	0.736	12,536.042	12,821.612	12,637.399	351.809 (*p* = 0.041)	356.043 (*p* = 0.040)	38%; 36%; 14%; 12%
5 Profiles	−6,038.898	0.794	12,217.796	12,562.450	12,340.124	338.175 (*p* = 0.001)	342.246 (*p* = 0.001)	35%; 32%; 16%; 13%; 4%
6 Profiles	−5,950.791	0.789	12,065.581	12,469.319	12,208.880	174.119 (*p* = 0.908)	176.215 (*p* = 0.906)	35%; 30%; 15%; 12%; 4%; 4%

*N* = 1,061. LL, Log Likelihood; AIC, Akaike Information Criteria; BIC, Bayesian Information Criteria; SABIC, sample-adjusted BIC; LMR-LRT, Lo–Mendell–Rubin likelihood ratio test; VLMR-LRT, Vuong-Lo–Mendell–Rubin likelihood ratio test.

### 3.3. Preschool children’s profiles

**Figure 1:**
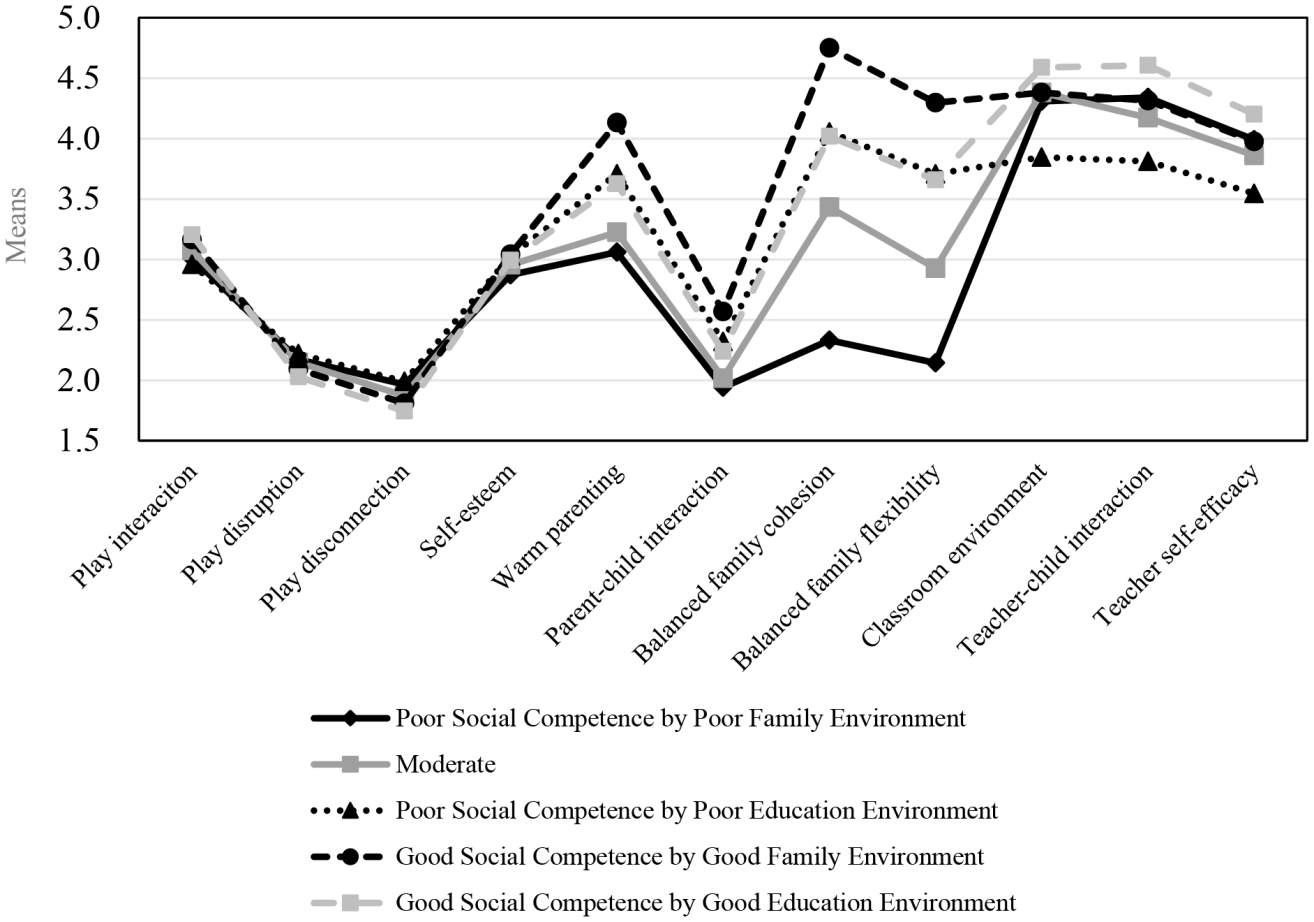
Preschool children’s bioecological profiles: final five-profile model (*N* = 1,016).

Mplus provides means and variances for each of the 11 indicator variables for each profile. The means from the best-fitting 5-profile model are depicted in Figure 1. The “Good Social Competence by Good Educational Environment” profile (*n* = 356, 35% of the sample) reflects preschool children who are higher in social competence and educational environment. The “Good Social Competence by Good Family Environment” profile (*n* = 132, 13%) includes those who are higher in social competence and familial environment. The “Moderate” profile (*n* = 162, 16%) includes those who are moderate in social competence, familial environment, and educational environment. The “Poor Social Competence by Poor Educational Environment” profile (*n* = 325, 32%) includes preschool children who are lower in social competence and educational environment. Finally, the “Poor Social Competence by Poor Familial Environment” profile (*n* = 41, 4%) includes preschool children who are lower in social competence and familial environment. In addition, this study conducted the posterior probabilities of membership to examine the likelihood of assignment to a profile. Posterior probabilities range from zero to one, with higher values representing a greater likelihood of correct assignment to a profile; posterior probabilities of more than 70% indicate profile membership confidence (Nagin, 1999). The posterior probabilities for the profiles’ membership were all higher than this value, ranging from 0.82 to 0.97, and the standard errors of the class-specific accuracy were lower than 0.01.

### 3.4. Association between preschool children’s profiles and school adjustment, academic performance, and executive function in first grade

Associations between preschool children’s bioecological profiles and their school adjustment, academic performance, and executive function in first grade are reported in Table 3.

**Table 3:** Multilevel regression coefficients for predicting school adjustment, academic performance, and executive function.

		School adjustment	Academic performance	Executive functions
		β (*SE*)	β (*SE*)	β (*SE*)
Step 1	Intercept	3.50^***^ (0.14)	3.47^***^ (0.17)	2.53^***^ (0.08)
	Covariates
	^a^Child’s gender	−0.37^***^ (0.04)	−0.33^***^ (0.05)	−0.25^***^ (0.02)
	^b^Maternal education level	0.06^*^ (0.02)	0.12^***^ (0.03)	0.04^*^ (0.01)
	^c^Subjective family SES	0.05^**^ (0.02)	0.06^**^ (0.02)	0.01 (0.00)
Step 2	Profile membership
	Good social competence by Good family environment	0.23^**^ (0.08)	0.28^**^ (0.10)	0.11^*^ (0.05)
	Good social competence by Good education environment	0.14^*^ (0.06)	0.05 (0.08)	0.09^**^ (0.04)
	Poor social competence by Poor family environment	0.09 (0.13)	−0.11 (0.16)	−0.03 (0.07)
	Poor social competence by Poor education environment	0.07 (0.06)	0.04 (0.08)	0.02 (0.04)
ΔR^2^		0.10^***^	0.08^***^	0.13^***^

^a^Child’s gender (0 = girl, 1 = boy), ^b^Maternal education level (0 = uneducated to 7 = graduate school graduate), ^c^Subjective family socioeconomic status (1 = lowest to 10 = highest), and Moderate profile specified as the reference group. ^*^p < 0.05, ^**^p < 0.01, and ^***^p < 0.001.

Based on the MANCOVA results, statistically significant differences were found between the profiles in terms of school adjustment (*p* = 0.013), academic performance (*p* = 0.010), and executive function (*p* = 0.014). However, caution is advised when interpreting these findings due to violations of the assumptions of homogeneity of covariance matrices and homoscedasticity, as indicated by Box’s M Test (*p* < 0.001) and Levene’s Test results (school adjustment, *p* = 0.011; academic performance, *p* = 0.002; executive function, *p* < 0.001).

All tolerance values were greater than 0.1, ranging from 0.78 to 1.00, and variance inflation factors (VIF) were less than 10, ranging from 1.01 to 1.28, indicating no issues of collinearity.

The unstandardized regression coefficients for the multilevel model predicting school adjustment indicated that the “Good Social Competence by Good Familial Environment” (*b* = 0.28, SE = 0.08, *p* < 0.01) and “Good Social Competence by Good Educational Environment” groups (*b* = 0.14, SE = 0.06, *p* < 0.05) reported significantly higher scores than the “Moderate” group (ΔR^2^ = 0.10). For academic performance, the “Good Social Competence by Good Familial Environment” group (*b* = 0.28, SE = 0.10, *p* < 0.01) scored higher than the “Moderate” group (ΔR^2^ = 0.08). For executive function, the “Good Social Competence by Good Familial Environment” (*b* = 0.11, SE = 0.05, *p* < 0.05) and “Good Social Competence by Good Educational Environment” groups (*b* = 0.09, SE = 0.04, *p* < 0.01) scored higher than the “Moderate” group (ΔR^2^ = 0.13).

## 4. Discussion

The importance of early school adjustment, academic performance, and executive function has received significant attention in both research and education communities. The numerous variables that predict early school adjustment and performance can be largely classified into individual and environmental characteristics. Many scholars support the fact that these two cannot act separately but interact with each other. However, they mostly used a variable-driven approach that is unsuitable for capturing interactions (Laursen and Hoff, 2006). To more appropriately capture the interaction between personal characteristics and the environment of preschoolers, the current study adopted a person-centered rather than a variable-centered approach. This study included a total of 11 indicators, comprising 4 for personal characteristics, 4 for familial environment, and 3 for preschool educational environment in the latent profile survey.

The results revealed profiles of preschool children with heterogeneous social competence, familial environment, and preschool educational environment. These profiles were also related to school adjustment, academic performance, and executive function in the first grade. This demonstrates that the familial environment during preschool age is consequential for school adjustment and performance in the first grade, while emphasizing the important role of parents and early childhood education teachers in children’s school success.

### 4.1. Bioecological profiles of preschool children

Preschool children exhibited five bioecological profiles: (1) Good Social Competence by Good Educational Environment, (2) Good Social Competence by Good Familial Environment, (3) Moderate (Social Competence by Environment), (4) Poor Social Competence by Poor Educational Environment, and (5) Poor Social Competence by Poor Familial Environment. These five bioecological profiles displayed clear relative differences in environmental characteristics rather than individual characteristics. Although all 11 indicators showed differences by profile, the profile decision was influenced by the order of familial environment, preschool educational environment, and individual social competence indicators. As reported in Figure 1, the difference in individual characteristics was insignificant compared to environmental characteristics when determining the profile. In other words, the bioecological profile reveals that the difference in environmental support is more obvious than that in individual characteristics during preschool years. This is in line with previous studies on the development and stability of executive function of preschoolers in Japan. Developmental changes in executive function are facilitated by both individual and environmental impacts, but stability in executive function is caused by environments such as parental interaction skills (Fujisawa et al., 2017). Some studies in Western countries acknowledged individual characteristics but emphasized the significant role that environmental factors play in promoting school adjustment, executive function, and academic performance. However, these studies did not directly compare the effects of environmental support and individual characteristics (Ansari and Purtell, 2017; Niklas and Schneider, 2017).

The profile group ratio was in the order of “Good Social Competence by Good Educational Environment,” “Poor Social Competence by Poor Educational Environment,” “Moderate (Social Competence by Environment),” “Good Social Competence by Good Familial Environment,” and “Poor Social Competence by Poor Family Environment.” It is a positive and encouraging result that, among the five groups, the “Good Social Competence by Good Education Environment” group accounts for the largest proportion. However, the fact that the ratio is only 3% different from the “Poor Social Competence by Poor Education Environment” group suggests that practical efforts by the Korean government and local communities are needed to prevent polarization of the educational environment in Korean society.

### 4.2. Relationship between the bioecological profile of preschoolers and early school adjustment and performance

Multilevel regression analyses revealed that there are significant differences between the distinct bioecological profiles of preschoolers and their school adjustment, academic performance, and executive function a year later. After controlling for child gender, maternal education level, and socioeconomic status based on previous studies, the regression analysis revealed that the “Good Social Competence by Good Familial Environment” group scored the highest in school adjustment, academic performance, and executive function a year later. The “Good Social Competence by Good Educational Environment” group scored higher than the moderate group in terms of school adjustment and executive function, but scored less than the “Good Social Competence by Good Familial Environment” group.

These results mean three things. First, when preschool children with good social competence receive good environmental support, their level of school adjustment and executive function is higher than others after a year. This result is in line with previous research that demonstrated that more risk factors were associated with less adaptive outcomes (Appleyard et al., 2005; Lanza et al., 2010). It indicated that the accumulation of more positive ecological factors exhibited more adaptive results. This fact implies the importance of accumulating various positive factors that affect children’s school adjustment and cognitive development.

Second, the “Good Social Competence by Good Familial Environment” group can be identified as one providing the strongest protective effect compared to other groups. In terms of school adjustment and executive function, it was higher than that of the moderate group as well as the “Good Social Competence by Good Educational Environment” group. The academic performance was also higher than that of the moderate group. This is consistent with previous studies that emphasized the importance of the preschoolers’ familial environment above all else in early school adjustment and performance. According to Pianta et al. (1997), both preschool child–mother and child-teacher interaction qualities predicted positive performance on child development measurements, but the quality of the former was more strongly related to school adjustment than that of the latter. Connell and Prinz (2002) emphasized that parent–child interactions were positively related to preschoolers’ school preparation, social skills, and communication skills, but their time at preschool educational institutions had mixed results. Buyse et al. (2011) established that the impact of teacher-child interactions on preschoolers may be limited if they already have a positive relationship with their parents. The educational environment can compensate for negative risk factors when preschoolers’ familial environment is weak. However, children who already have a good familial environment are more likely to form a positive internal working model based on their experience with early attachments, so they may be less affected by the preschool educational environment in school adjustment (Dykas et al., 2014). In addition, the positive aspects of the familial environment tend to remain stable over time (Dallaire and Weinraub, 2005; Matte‐Gagné et al., 2013). Unlike the educational environment, a good familial environment during the preschool period is maintained consistently during school one year later, which may have positive effects on children’s school adjustment and cognitive development stability. In addition, similar to other Asian parents, Korean parents exhibit responsive parenting by paying attention to their children’s educational needs, and this cultural characteristic of familial environment support may have had a strong impact on school adjustment, executive function, as well as academic performance (Cheah et al., 2015; Kim and Chung, 2017).

Third, the “Good Social Competence by Good Educational Environment” group also achieved superior scores compared to the moderate group in terms of school adjustment and executive function. This result supports many previous studies and policy programs that emphasize the importance of the preschool educational environment (Magnuson et al., 2004; Yoshikawa et al., 2013). However, there was no significant difference between the “Good Social Competence by Good Educational Environment” group and the moderate group in terms of academic performance. According to Hamre and Pianta (2001), the quality of teacher-child relationships is a stronger predictor of behavior than academic outcomes. In this study, the educational environment characteristics included teacher-child interaction indicators, which affected behavioral outcomes such as school adjustment and executive function, but might not have influenced academic performance, which is more objective and less affected by teacher-child relationship quality.

### 4.3. Limitations and future directions

The limitations of this study need to be noted as they present important directions for future research.

First, data collection on child–parent interactions, family interactions, and child-teacher interactions was conducted using a self-report format. To avoid potential socially desirable effects in future studies, various measurement methods, such as observations recorded by data collectors, should be adopted.

Second, the effect sizes of this study were small. In many social science studies, small effect sizes still reflect meaningful differences that can inform early childhood education programs (Cortina and Landis, 2009); however, the results need to be interpreted carefully, as various profiles and school adjustment, academic performance, and executive function are not necessarily causal.

Third, child temperament characteristics were not included in the index. While indicators such as preschoolers’ peer interaction and self-esteem may best represent the individual characteristics of a 5-year-old, they may more clearly explain the child’s interaction with its environment if they include more strongly related indicators such as the child’s temperament.

Fourth, although beyond the scope of this study, the degree of individual by environmental interaction may vary by race and culture. Future research can employ panel data from more diverse countries to compare the commonalities and differences between countries in how preschoolers’ individual, familial, and environmental characteristics are combined to form identified groups, and how they influence school adjustment and cognitive development.

## 5. Conclusions and implications

School psychologists have emphasized the need for rigorous empirical research that examines various contexts affecting school adjustment, academic performance, and executive function (Goble et al., 2019). Our results support the concept that despite its potential limitations, not only do children’s personal characteristics matter, but their family and preschool educational environments also have a complex influence on school adjustment. This is particularly important in terms of school and developmental psychologists’ roles in promoting school adjustment and academic preparation for elementary school children. Efforts to prevent academic maladjustment and lack of performance can be most effective when addressing problems of family and preschool education together rather than individually. Given studies suggesting that preschool children’s familial environment is a strong predictor of subsequent school preparation (Niklas and Schneider, 2017; Skibbe et al., 2019), programs dealing with child-rearing and family climate in preschool facilities can be particularly effective in promoting early school adjustment skills. This study has implications for developing (1) programs for preschoolers’ families to support early school adjustment and performance, and (2) policies to ensure no significant differences in the educational environment for preschoolers.

## Data availability statement

Publicly available datasets were analyzed in this study. This data can be found here: https://panel.kicce.re.kr/pskc/module/rawDataManage/index.do?menu_idx=56.

## Ethics statement

Ethical review and approval was not required for the study on human participants in accordance with the local legislation and institutional requirements. Written informed consent to participate in this study was provided by the participants’ legal guardian/next of kin.

## Author contributions

Y-EL confirms being the sole contributor of this work and has approved it for publication. The author conducted all aspects of the research including the conceptualization and design of the study, data collection and analysis, and writing and revising of the manuscript.

## Funding

This work was supported by the Gachon University research fund of 2021 (GCU- 202102900001).

## Conflict of interest

The author declares that the research was conducted in the absence of any commercial or financial relationships that could be construed as a potential conflict of interest.

## Publisher’s note

All claims expressed in this article are solely those of the authors and do not necessarily represent those of their affiliated organizations, or those of the publisher, the editors and the reviewers. Any product that may be evaluated in this article, or claim that may be made by its manufacturer, is not guaranteed or endorsed by the publisher.
